# Stage-specific transcription during development of *Aedes aegypti*

**DOI:** 10.1186/1471-213X-13-29

**Published:** 2013-07-22

**Authors:** Brent W Harker, Susanta K Behura, Becky S deBruyn, Diane D Lovin, Akio Mori, Jeanne Romero-Severson, David W Severson

**Affiliations:** 1 Department of Biological Sciences, Eck Institute for Global Health, University of Notre Dame, Notre Dame, IN 46556, USA

**Keywords:** Development, Diptera, Gene expression, Aedes aegypti, Microarray, Transcript, Holometabolous

## Abstract

**Background:**

*Aedes aegypti* is the most important global vector of dengue virus infection in humans. Availability of the draft genome sequence of this mosquito provides unique opportunities to study different aspects of its biology, including identification of genes and pathways relevant to the developmental processes associated with transition across individual life stages. However, detailed knowledge of gene expression patterns pertaining to developmental stages of *A. aegypti* is largely lacking.

**Results:**

We performed custom cDNA microarray analyses to examine the expression patterns among six developmental stages: early larvae, late larvae, early pupae, late pupae, and adult male and female mosquitoes. Results revealed 1,551 differentially expressed transcripts (DETs) showing significant differences in levels of expression between these life stages. The data suggests that most of the differential expression occurs in a stage specific manner in *A. aegypti.* Based on hierarchical clustering of expression levels, correlated expression patterns of DETs were also observed among developmental stages. Weighted gene correlation network analysis revealed modular patterns of expression among the DETs. We observed that hydrolase activity, membrane, integral to membrane, DNA binding, translation, ribosome, nucleoside-triphosphatase activity, structural constituent of ribosome, ribonucleoprotein complex and receptor activity were among the top ten ranked GO (Gene Ontology) terms associated with DETs. Significant associations of DETs were also observed with specific KEGG (Kyoto Encyclopedia of Genes and Genomes) pathway modules. Finally, comparisons with the previously reported developmental transcriptome of the malaria vector, *Anopheles gambiae*, indicated that gene expression patterns during developmental processes reflect both species-specific as well as common components of the two mosquito species.

**Conclusions:**

Our study shows that genes involved in the developmental life cycle of *A. aegypti* are expressed in a highly stage-specific manner. This suggests that transcriptional events associated with transition through larval, pupal and adult stages are largely discrete.

## Background

Mosquito (Culicidae) development, as characteristic of all holometabolous insects, proceeds through embryonic, larval, pupal, and adult stages that reflect considerable morphological and physiological differences. These stages also exhibit distinct niche partitioning as larvae and pupae are aquatic while adults are free-flying and terrestrial. In addition, following an estimated ~192-230 million years of divergence among the major mosquito lineages [1], it is anticipated that individual species might have evolved in molecular pathways of developmental processes as seen throughout the evolution of insect metamorphosis [2]. Larvae of all mosquito species progress through four instars that include periods of continuous growth interrupted by shedding of the old cuticle or ecdysis [3,4]. The molting process begins with physical separation of the epidermis from the old endocuticle, a process known as apolysis. In response to hormonal changes by increasing their rate of protein synthesis during this period, the epidermal cells secrete a lipoprotein that forms the cuticulin layer to insulate and protect them from the molting fluid's digestive action. The cuticulin layer becomes part of the new exoskeleton's epicuticle. When the new exoskeleton is ready, the old exoskeleton splits open. Ecdysis (shedding old exoskeleton) continues to fully expand the new exoskeletons. After ecdysis, sclerites harden and darken within the exocuticle, the process known as sclerotization, which gives the exoskeleton its final texture and appearance. With the completion of the four instars of larval molting and sclerotization, metamorphosis, the transformation from larvae to pupae to adult stages, begins. It includes complex processes that involve larval and pupal tissue histolysis and remodeling leading to adult tissue formation. The cascades of transcriptional events associated with insect ecdysis and metamorphosis are controlled by coordinated ecdysteroid and juvenile hormone (JH) activities [5-7].

The mosquito, *Aedes aegypti*, is the principal global vector for dengue viruses. Dengue fever (DF) is caused by infection with dengue virus throughout the subtropics and tropics, with >2.5 billion people at risk. An annual incidence of ~50 million cases and ~500,000 cases of dengue hemorrhagic fever (DHF) and dengue shock syndrome (DSS) results in ~24,000 deaths per year [8-11]. No effective vaccines are currently available and no drug treatments exist. Thus mosquito control remains the most effective strategy for controlling dengue and other mosquito-borne diseases, in spite of resistance to insecticides in specific populations [12]. *A. aegypti* maintains a strong association with humans, breeding in virtually any container that holds water long enough for larval/pupal development [13], and because of a strong dietary preference for human blood [14] it is capable of completing the entire life cycle within human dwellings.

To date, gene expression studies pertaining to *A. aegypti* development are limited [15-21]. In the malaria vector mosquito *Anopheles gambiae*, microarray studies have been performed to study life cycle developmental transcriptome [22,23]. These independent studies identified a total of 1,571 [22] and 560 [23]*A. gambiae* transcripts, respectively, that showed differential regulation specific to development. Comparative global expression analyses with *Drosophila melanogaster* revealed a strong positive correlation of development-related expression between orthologous genes [22]. However, a genome-scale transcriptional analysis of *A. aegypti* life cycle development is lacking.

A draft whole genome sequence is available for *A. aegypti*[18]. As part of the genome sequencing effort a large collection of expressed sequenced tags (ESTs) derived from a broad range of tissues and strains was generated. Here we employed a custom cDNA-based microarray platform that represents 9,504 unique EST contig assemblies. We compared transcriptional profiles across the *A. aegypti* life cycle including early and late larvae, early and late pupae, mixed adults, and adult males and females. Where possible, we also compared our results for *A. aegypti* with those previously reported for similar stages in *A. gambiae*.

## Methods

### Ethics statement

This study was performed in accordance with the recommendations in the Guide for the Care and Use of Laboratory Animals of the National Institutes of Health. The animal use protocol was approved by the University of Notre Dame Institutional Animal Care and Use Committee (Study #11-036).

### Mosquitoes

*Aedes aegypti* Liverpool IB-12 strain was reared at 26°C with 84% relative humidity and in a 16-h light/8-h dark cycle with 1-h crepuscular periods. Larvae were reared on a bovine liver powder (MP Biomedical) suspension as the food source and adults were provided a 5% sugar solution *ad libitum*. The larval density was 500 per 1,500 cm^3^ in all the rearing to prevent crowding effects. The detailed protocol on rearing and maintenance of *A. aegypti* is provided elsewhere [24].

### RNA preparation

**Figure 1:**
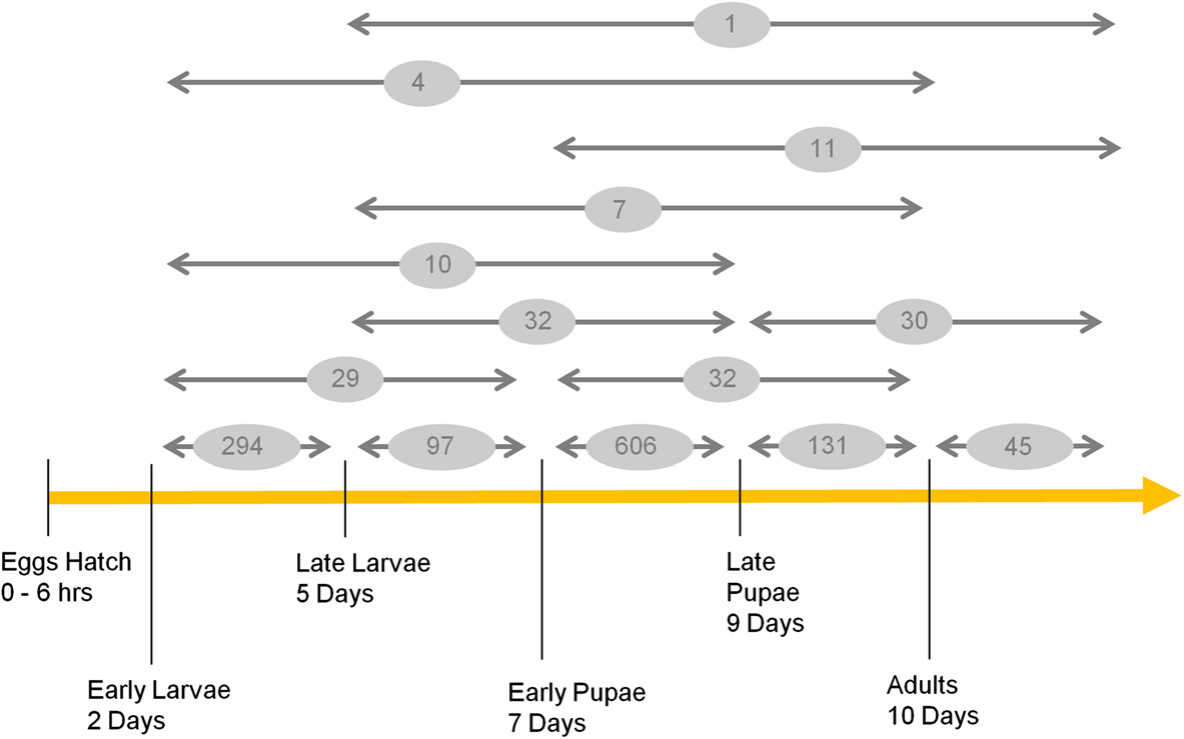
**Approximate time lines of different developmental stages for *A. aegypti.*** The numbers of differentially expressed genes (DETs) associated with specific developmental stages are shown by horizontal arrows. The developmental periods compared in the study are shown. Egg hatch occurred within an ~6 h window. The early stage larvae primarily represented L2 stage larvae with some L3 stage. The late-stage larvae represented 5th day post egg hatch and were all L4 stage. The early pupae stage was day 7 when newly emerged pupae (<2 h) were collected. The late pupae stage was just before adult eclosion (~ within 2 h post tanning) on the 9^th^ day. The 10^th^ day after egg hatch represented the first day of adult emergence.

Total RNA was extracted from early larvae, late larvae, early pupae, late pupae, and adult male and female mosquitoes using TRIzol (Invitrogen) according to the manufacturer’s protocol. These developmental stages represented the time duration (in days) after egg hatching as shown in Figure 1. Egg hatch was completed within ~6 h post-immersion in water. Most of the early stage larvae collected on day 2 after eggs hatched were L2 stage with a few L3 stage. The late stage larvae period represented 5 days post egg hatch and these were all L4 stage. At day 7, pupae (mixed sexes) were collected within 2 h following pupation. Just before adult eclosion (~ within 2 h), the late pupae (post-tanning and mixed sexes) were collected. The 10th day after egg hatch represented the first day of adult emergence (mixed sexes). Comparison of larvae, pupae and adult stages between *A. aegypti* and *A. gambiae* is empirical without referring to specific developmental features of the species. Comparison of empirical developmental stages between *A. gambiae* and *D. melanogaster* is an established method as reported in the study by Koutsos *et al*. [22]. Approximately 20 individuals from the various developmental stages were used for the extractions. Following extraction, the RNA was treated with 1.0 unit of DNase I (Invitrogen) according to manufacturer’s instructions. First strand cDNA synthesis and labeling was performed using 15 μg of total RNA using the Genisphere 3DNA® Array 50 kit (Genisphere) for each dye, cyanine 3 (Cy3) and cyanine 5 (Cy5), according to the manufacturer’s protocol. Three biological replicates were prepared for each stage.

### Microarray content and design

Custom microarrays were generated from 9,504 unique cDNA amplicons as previously described [25]. The cDNAs were obtained from a number of *A. aegypti* strains and included tissue-specific and pathogen response-specific origins generated as part of the genome sequence annotation effort [18]. Consensus EST assemblies and associated cDNA clones were downloaded at the *A. aegypti* Gene Index [26].

### Microarray hybridization and analysis

Five developmental comparisons were investigated: 1) early larvae-late larvae, 2) late larvae-early pupae, 3) early pupae-late pupae, 4) late pupae-adult mixture, and 5) adult male-adult female. Hybridization experiments were carried out following the two step protocol as recommended by the manufacturer (Genisphere). All of the hybridization comparisons included one dye-swap in order to eliminate dye fluorescence bias. The entire experiment was performed with a total of three biological replicates. After hybridization and washing, the microarray slides were scanned at two wavelengths, 532 and 635 nm, using the GenePix Pro 4200A scanner (Molecular Devices Corp).

Spot intensity data was quantified using the segmentation and data analysis software GenePix Pro 6.0 (Molecular Devices Corp). The average signal intensities were normalized with an intensity dependent (Lowess) normalization using GeneSpring GX 7.3 software (Agilent). Statistical analysis of the data was conducted using Significance Analysis of Microarrays (SAM) [27]. All the raw as well as processed expression data of the microarray experiments have been deposited in ArrayExpress under the accession number E-TABM-385.

Annotation information and gene ontology (GO) data for the transcripts were obtained at VectorBase [28]. Fisher’s exact test was used to determine significant associations of GO terms with the differentially expressed genes. The numbers of significant and non-differentially expressed transcripts (DETs) associated with each GO term were compared with the respective counts of genes with all other GO terms for the entire gene set. Similar comparisons were also made for GO terms associated with DETs for each developmental stage. Association of the DETs with *A. aegypti* pathways was determined at KEGG [29]. All statistical tests were conducted using the statistical analysis package R [30]. The modular expression patterns were predicted by weighted gene correlation network analysis of DETs using default parameters with the WGCNA program [31]. The expression fold-changes of transcripts among the five pairs of developmental stages were clustered using hierarchical clustering method (average linkage) implemented in Cluster 3.0 software [32]. The rank order correlation of fold-changes was used to determine clusters among genes (columns) and stages (rows). The clusters were viewed by the TreeView program (http://www.eisenlab.org/eisen/).

### Quantitative real-time PCR analysis

Expression levels of a randomly selected set of genes were measured using quantitative real-time PCR (qRT-PCR) analysis using SYBR Green dye technology (Applied Biosystems). Primer Express Software version 3.0 (Applied Biosystems) was used to design primers (Additional file 1). All amplifications and fluorescence quantification were performed using an ABI 7500 Fast System Sequence Detector System (Applied Biosystems) and the Sequence Detector Software version 1.3 (Applied Biosystems). The reactions were performed in a total volume of 25 μl containing 12.5 μl of SYBR Green PCR Master Mix, 10 ng of cDNA (the same samples used in microarrays), 300 nmol of each primer, and nuclease free water. Reactions were performed with the following conditions: 50°C for 2 min, 95°C for 10 min followed by 40 cycles of denaturation at 95°C for 15 s, annealing and extension at 60°C for 1 min. Three biological replicates were performed for each assay. Melting curves of the data points were used to determine the specificity of the PCR reaction. Data was used from assays only when PCR efficiency was greater than 95%. Expression values were obtained by using the delta-delta cycle threshold (∆∆C_T_) method [33] using the *ribosomal protein S17* (*RpS17*) gene as the reference control [34].

### Comparative analysis

The developmental transcriptome data of *A. gambiae* from a previous study [22] were used for comparisons with our current microarray data for *A. aegypti*. The *A. gambiae* microarray expression data were downloaded at VectorBase [28] and were compared with DETs at five life stages (Lb, Le, P, M, F) of *A. gambiae*[22]. The different life stages of *A. gambiae* were represented as La, Lb, Lc, Ld and Le stages for larvae whereas P, M and F represented the pupae, male- and female- adults stages in that study [22]. For comparison with *A. aegypti*, the specific stages were chosen that generally approximated to early larvae, late larvae, early pupae and adult male and female stages of *A. aegypti*. Our objective of this analysis was to detect if larvae, pupae and adult stages of both the mosquitoes have signature gene expression patterns, as it is difficult to ascertain that the chosen developmental times corresponded to the same exact life stages of both the species. A total of three comparisons (Lb-Le, Le-P, and M-F) between the *A. gambiae* data and our data for *A. aegypti* were performed. The fold change of gene expression levels were compared between the two data sets. The orthologous genes between the two species were obtained from Biomart data included in VectorBase [28]. Only genes that were 1-to-1 orthologs were considered (n = 8,325), and from these the list of genes expressed in both organisms in the similar developmental stages were identified.

## Results

### Identification of differentially expressed transcripts related to development

The DETs were determined at five developmental stages of *A. aegypti*: early larvae – late larvae (EL-LL), late larvae – early pupae (LL-EP), early pupae – late pupae (EP-LP), late pupae – adult male and female mix (LP-AdultMix) and adult male – adult (AM-AF). The significance levels of differential expression for each comparison were assessed by SAM analysis where the significance threshold (δ) ranged within 0.34 to 0.51, while the false discovery rate ranged from 4.9 to 5.5%. The minimum significant fold change was 1.9 for the five comparisons. To validate the microarray data, expression patterns for nine randomly selected genes were determined using qRT-PCR. The results revealed highly similar trends between qRT-PCR and microarray data for the expression levels of the genes (Additional file 2).

Data analyses indicated that 1,551 cDNAs were significantly differentially expressed at the different stages of development of *A. aegypti* (Table 1). Most of the differentially expressed transcripts (n=1173, amounting to 75.6% of all significant transcripts) were stage-specific (Figure 1). The genes were differentially expressed between specific developmental times such as early larvae vs. late larvae, late larvae vs. early pupae, early pupae vs. late pupae and male vs. female adults as shown in Figure 1. The pupal stage involved a greater number of DETs compared to any other stage of development. The transition of early pupae to late pupae involved 606 transcripts (~ 40% of all the detected DETs). A total of 294 unique transcripts were differentially expressed between early and late larval stages thus representing the second most dynamic transcriptional period of *A. aegypti* development. However, transitioning from larval to pupal stage involved fewer genes as only 97 DETs (less than one third the number of different transcripts expressed in the larvae) were found significant at this period of development. Similarly, transitioning from pupal to adult stage was associated with 131 DETs, which is 4.6-fold less than the number of different transcripts expressed between early and late pupal stages. These larval-to-pupal and pupal-to-adult stage transitions were associated with only 6.2% or 8.4% of all significant DETs, respectively. On the other hand, only 378 DETs showed significant differential expression at more than one developmental time. Based on comparisons across developmental times, 25 different multiple-stage expression patterns were identified (Table 2). Although many of the DETs listed in Table 1 have been annotated as protein coding genes as annotated from genome sequences of *A. aegypti* (Additional file 3), a number of these transcripts are not represented in either the official gene set AaegL1.2 or NCBI databases (indicated as “#N/A”). The list of VectorBase annotated genes of the ESTs showing differential expression at more than one developmental time (Table 2) is also provided in Additional file 4 along with the life stages at which these genes are differentially expressed. Two of them, indicated as “#N/A” in Additional file 4 are however not represented in either the official gene set AaegL1.2 or NCBI databases.

**Table 1:** Numbers of significant DETs identified from the microarray analysis

**Developmental stages**	**Significant DETs**
EL-LL	294
LL-EP	97
EP-LP	606
LP-AdultMix	131
AM-AF	45
Non-specific	378

**Table 2:** **Different patterns of differentially expression of transcripts where significant changes in expression level are evident in multiple developmental stages of*A. aegypti***

**Expression pattern**	**No. of transcripts**	**Developmental stages investigated**
1	29	EL-LL + LL-EP
2	55	EL-LL + EP-LP
3	25	EL-LL + LP-AdultMix
4	25	LL-EP + LP-AdultMix
5	32	EP-LP + LL-EP
6	77	EP-LP + LP-AdultMix
7	11	AM-AF + EL-LL
8	8	AM-AF + LL-EP
9	12	AM-AF + EP-LP
10	30	AM-AF + LP-AdultMix
11	10	EL-LL + LL-EP + LP-AdultMix
12	4	EL-LL + EP-LP + LL-EP
13	11	EL-LL + EP-LP + LP-AdultMix
14	7	EP-LP + LL-EP + LP-AdultMix
15	2	AM-AF + EL-LL + LL-EP
16	5	AM-AF + EL-LL + EP-LP
17	3	AM-AF + EL-LL + LP-AdultMix
18	5	AM-AF + LL-EP + LP-AdultMix
19	1	AM-AF + EP-LP + LL-EP
20	11	AM-AF + EP-LP + LP-AdultMix
21	4	EL-LL + EP-LP + LL-EP + LP-AdultMix
22	2	AM-AF + EL-LL + LL-EP + LP-AdultMix
23	3	AM-AF + EL-LL + EP-LP + LL-EP
24	5	AM-AF + EL-LL + EP-LP + LP-AdultMix
25	1	AM-AF + EP-LP + LL-EP + LP-AdultMix

### Correlated expression patterns of DETs

**Figure 2:**
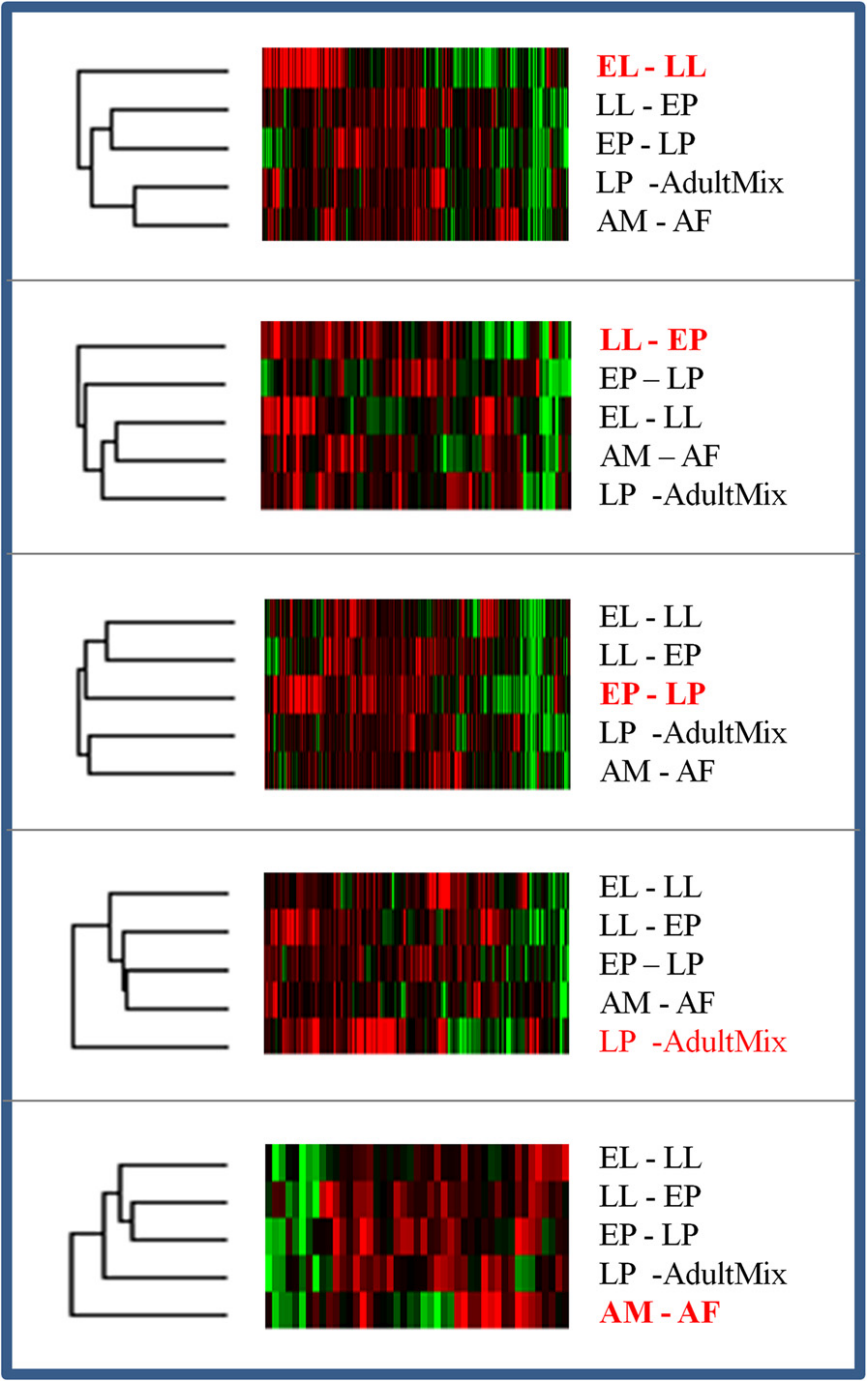
**Hierarchal cluster analysis of DETs among different stages of development of *A. aegypti.*** The pairwise stages at which differential expression was determined are shown to the right of each self-organizing expression map generated by Cluster 3.0. The stages highlighted in red text indicate the developmental times where the DETS are significantly differentially expressed in *A. aegypti*. Red and green colors in the heat maps indicate up-regulated (fold-change range: 1.91 to 2.83) and down-regulated genes (fold-change range: -3.21 to - 1.93), respectively. The dark color indicates no significant changes in expression between stages and assumes value zero in the cluster analysis. The cluster trees for expression levels among the developmental stages are shown at the left of the corresponding maps.

Based on hierarchical clustering of gene expression levels, we observed evidence for highly correlated expression patterns of stage-specific DETs (Figure 2). For example, the transcripts which show significant differential expression between the early larval and late larval period (EL-LL) show lower correlated expression with the LL-EP and EP-LP stages or LP-AdultMix and the adult (AM-AF) stages. Similarly, the LL-EP stage specific DETs show lower correlated expression levels with the EL-LL, LP-AdultMix and the adult (AM-AF) stages. DETs specific to the pupal stages (EP-LP) show stronger correlated expression with early developmental stages (such as between EL-LL and LL-EP) than with the late stages (LP-AdultMix and AM-AF). The transcripts that are differentially expressed between pupal and adult stages (LP-AdultMix) show lower correlated expression levels with LL-EP, EP-LP as well as AM-AF. Finally, genes that are significantly differentially expressed between adult male and adult females show low correlated expression levels with the EL-LL, LL-EP and EP-LP stages. Thus the observed expression patterns of *A. aegypti* genes indicate that developmentally regulated genes are often activated or deactivated in a highly correlated manner from an early larval stage through adult eclosion.

**Figure 3:**
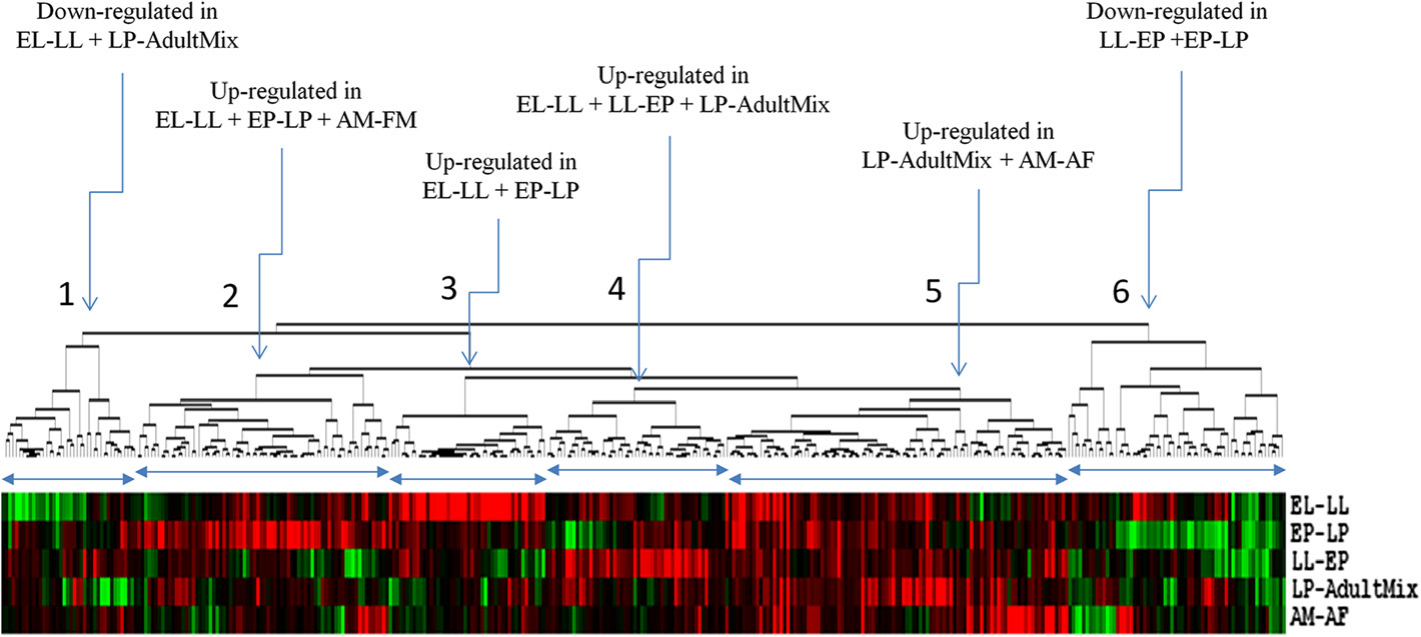
**DETs showing overlapping expression patterns of DETs.** Six cluster groups of expression wherein the subsets of DETs are either up-regulated or down-regulated among more than one developmental stage (see Figure 2 for heat map details). The six clusters are indicated by arrows pointing to the tree nodes for each expression group. The developmental stages are shown to the right of the self-organizing map.

**Figure 4:**
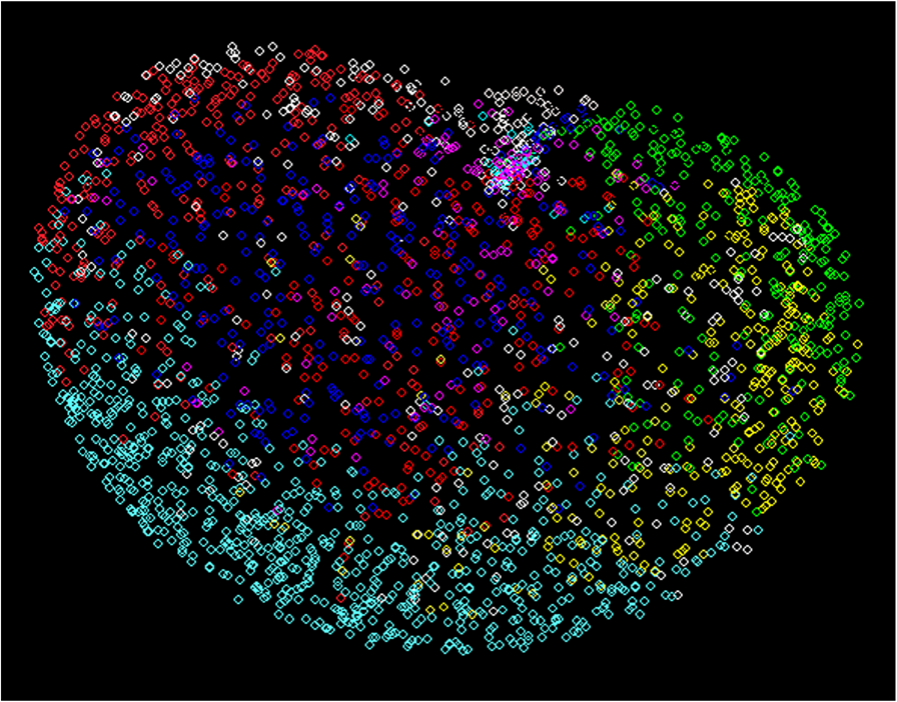
**Weighted gene correlation network analysis of DETs specifies modular patterns of gene networking among developmental stages.** Different colors identify DETs that represent different modules of expression. Each small square in the figure represents a differentially expressed transcript. The color codes of predicted modules correspond to different cluster patterns of expression shown in Figure 3 as follows: white color module - cluster 1, green color module – cluster 2, yellow color module – cluster 3, blue color module – cluster 4, red color module – cluster 5 and turquoise color module – cluster 6.

The hierarchical clustering of the transcripts (n=378) that showed significant differential expression at multiple time points during development revealed six primary clusters of gene expression patterns. Four of these clusters represented up-regulated genes and the other two clusters represented down-regulated genes (Figure 3). The down-regulated transcripts were mainly associated with the EL-LL + LP-AdultMix and EP-LP + LL-EP. The up-regulated genes were also associated with clusters wherein the patterns were common to two or three different stages and hence represent complex transcriptional activities during development. The overlapping of DETs among different developmental stages may reflect gene networks that intersect to form modules of genes with similar expression. This is further evident from weighted gene correlation network analysis [31] of these DETs where modular patterns of gene networking were apparent (Figure 4). The gene networking patterns, shown with different colors in Figure 4, reveal one-to-one correspondence with four of the six expression clusters shown in Figure 3. These results suggest that although developmental processes of *A. aegypti* involve largely stage-specific gene expressions, the 378 genes that show differential expression among different stages of development may be involved in cross-talking among stages.

### Gene annotation and functional assignments

The cDNAs were annotated by reciprocal blast analysis against gene build AaegL1.2 of *A. aegypti* at VectorBase [28]. The 1,551 significant DETs were found to represent a total of 927 annotated genes (1,225 gene transcripts) and these were associated with 2,503 GO terms. The difference between the number of significant DETs and the VectorBase annotated genes is possibly due to discrepancies in assembly methods of EST sequences versus gene annotation from the genome assembly [35]. Such difference between ESTs and annotated genes was also reported in an earlier study [22] while profiling gene expression of *A. gambiae*. In our current study, a total of 33 specific GO terms were significantly (*p* < 0.05) associated with the 1,551 DETs (Table 3). These GO terms were identified from all the DETs identified across all the developmental stages. Hydrolase activity, membrane, integral to membrane, DNA binding, translation, ribosome, nucleoside-triphosphatase activity, structural constituent of ribosome, ribonucleoprotein complex and receptor activity were among the top ten ranking GO terms associated with the differentially expressed genes. It was further observed that specific GO terms were differentially distributed among the DETs associated with different stages of *A. aegypti* development (Additional file 5). These GO terms were identified from the transcripts that were differentially expressed between specific developmental stages.

**Table 3:** **List of gene ontology (GO) terms significantly associated with the differentially expressed transcripts of *A. aegypti *during development**

**GO term**	**Genes**	**p-value**
Hydrolase activity	59	0.043693
Membrane	47	0.000346
Integral to membrane	44	0.000174
DNA binding	35	0.011245
Translation	25	0.025208
Ribosome	23	0.005415
Nucleoside-triphosphatase activity	21	0.050505
Structural constituent of ribosome	20	0.005092
Ribonucleoprotein complex	15	0.005773
Receptor activity	11	0.002999
Regulation of transcription, DNA-dependent	11	0.017637
Structural molecule activity	8	0.001117
Phosphoprotein phosphatase activity	6	0.028601
Cellular amino acid biosynthetic process	5	0.000204
Cellular iron ion homeostasis	4	0.007464
Ferric iron binding	4	0.00544
Iron ion transport	4	0.00544
Structural constituent of cuticle	4	0.000604
G-protein coupled receptor protein signaling pathway	3	0.000704
Intracellular membrane-bounded organelle	3	0.00751
Protein catabolic process	3	0.037129
Clathrin coat of coated pit	2	0.01173
COPI vesicle coat	2	0.01173
‘de novo’ IMP biosynthetic process	2	0.01173
Galactose metabolic process	2	0.035868
G-protein coupled receptor activity	2	0.006356
Methionine biosynthetic process	2	0.01173
Mitochondrial intermembrane space protein transporter complex	2	0.035868
Non-membrane spanning protein tyrosine phosphatase activity	2	0.004084
Protein import into mitochondrial inner membrane	2	0.035868
Pyrroline-5-carboxylate reductase activity	2	0.022467
Signal transducer activity	2	0.000381
Spermatogenesis	2	0.022467

The numbers of significantly expressed genes associated with the GO terms are shown. The Fisher’s exact test p-values are shown for significance association with the specific GO term.

**Figure 5:**
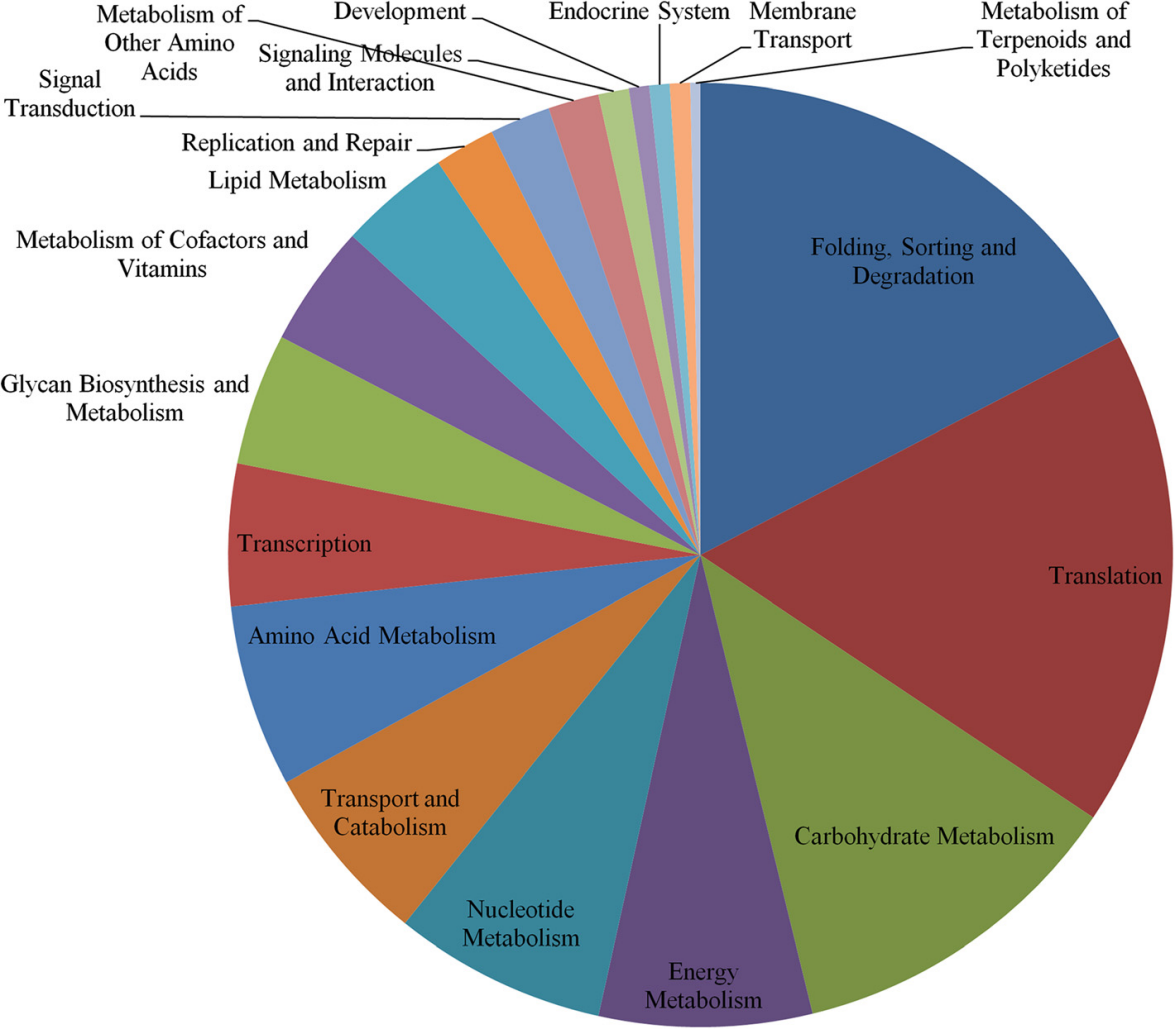
**Pie chart showing distribution of DETs among different KEGG pathways.** Different colors show different pathways.

In addition to gene ontology analysis, we also analyzed the 1,551 DETs for association with KEGG pathways predicted for *A. aegypti*. A total of 19 KEGG pathway modules were represented by these DETs (Figure 5). Of these, protein folding/sorting/degradation, translation and carbohydrate metabolism were identified as the top three ranking pathways based on transcripts differentially expressed at multiple developmental stages of *A. aegypti*. We wanted to know if genes related to specific pathways are differentially expressed at specific developmental stages of *A. aegypti*. Based on comparisons of numbers of genes differentially expressed at specific times and their association with KEGG pathways, it was observed that genes related to development and metabolism of amino acids pathways were associated with transcripts differentially expressed between early and late larval stages, whereas genes related to translation, transcription, carbohydrate metabolism, protein folding and sorting, transport and catabolism and glycan biosynthesis were associated with transcripts differentially expressed between early pupal and late pupal stages (Additional file 6). Transcripts differentially expressed between pupal and adult stages were significantly associated with energy metabolism and lipid metabolism related genes. Additionally, several genes related to dorso-ventral axis formation, notch signaling, neuroactive ligand-receptor interaction, hedgehog signaling and TGF-beta signaling pathways were identified wherein genes were differentially expressed at specific stages of *A. aegypti* development (Additional file 7).

### Comparison with developmental transcriptome of *Anopheles gambiae*

**Figure 6:**
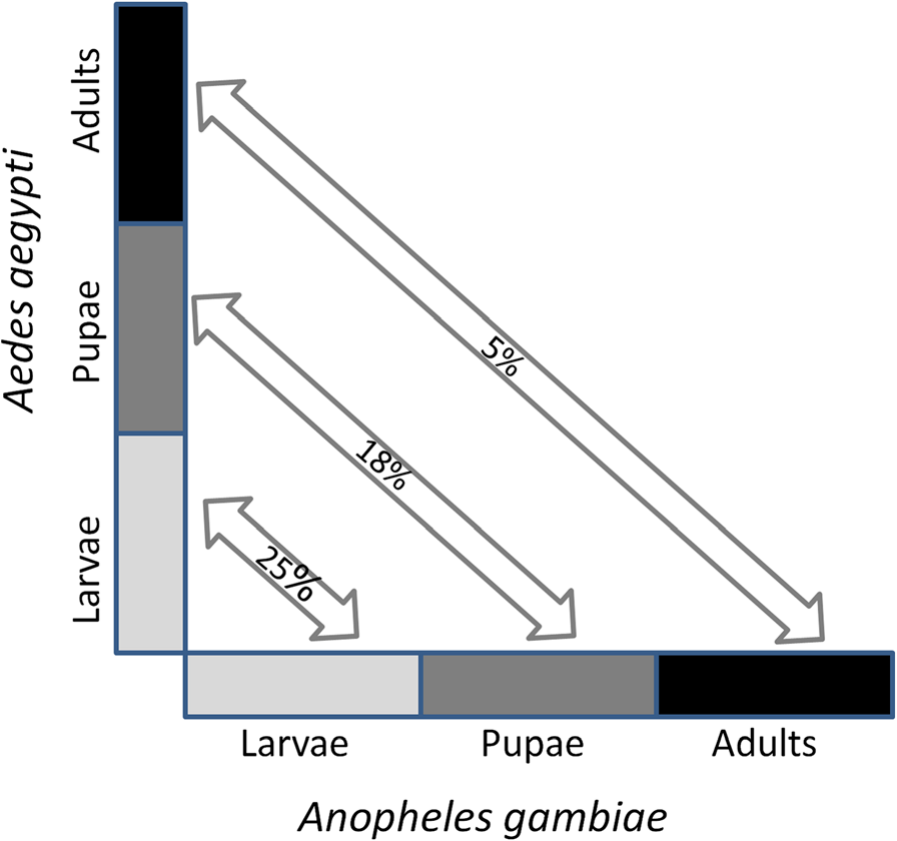
**Comparison of gene expression patterns that are conserved between *A. aegypti *and *A. gambiae.*** The percentages of one-to-one orthologs of *A. gambiae* identified in a previous study [22] with significant stage-specific expression (at larvae, pupae and adult stages) that also show significant expression at the same developmental stages of *A. aegypti* are listed.

Although developmental processes among mosquitoes may differ from species to species, a large number of orthologous genes known to play developmental functions are conserved among *A. aegypti*, *A. gambiae* and *Culex quinquefasciatus*[36]. This is also reflected from gene expression patterns by comparing our *A. aegypti* microarray data with previously reported microarray data for *A. gambiae*[22]. Although, Harker *et al.*[23] recently reported gene (n = 8,664) expression profiles of *A. gambiae* developmental stages, we chose to make comparisons with Koutsos *et al.*[22] data as this represented results for the complete annotated gene set. Three specific developmental stages were comparable to both data sets: 1) larval stage (Lb-Le stage of *A. gambiae* vs. EL-LL stage of *A. aegypti*), 2) late larval – early pupal stage (Le-P stage of *A. gambiae* vs. LL-EP stage of *A. aegypti*) and 3) adult stage (M-F of *A. gambiae* vs. AM-AF stage of *A. aegypti*). Of the differentially expressed genes at Lb-Le stage of *A. gambiae*, a total of 114 genes had one-to-one orthologs in *A. aegypti* that were also significant in our *A. aegypti* microarray data. However, only 29 of these were differentially expressed at the same developmental stage (EL-LL stage) of *A. aegypti*. The remaining 85 genes were expressed at other stages of development suggesting that only 25% of the orthologous genes associated with larval stage specific transcriptional activity of *A. gambiae* reflect similar changes in gene expression in the *A. aegypti* larval stages (Figure 6). These genes are mostly related to proteolysis, oxidative phosphorylation, protein biosynthesis, glutamine (amino acid) metabolism, and generic metabolism functions. Several of these genes (n = 12) show similar expression changes (fold-changes) between early- and late-larval stages of both species (Additional file 8) indicating that these genes may have role in larval development of both mosquitoes. The remaining genes (n = 17) showed differential changes between the two mosquitoes where they were up-regulated in one species but down-regulated in the other. Similarly, at the late larval-early pupal stage, a total of 82 differentially expressed genes represented orthologs between the two species but only 15 (18%) of these genes were differentially expressed at the same stage of development of *A. aegypti* (Figure 6). These common genes are related to proteolysis, coenzyme metabolism, protein modification, chromatin assembly and disassembly, carboxylic acid metabolism, and signal transduction functions. Similar to the larval stages, 8 of these 15 orthologous differentially expressed genes at the late larval-early pupal stage showed similar expression changes (fold-changes) in both species (Additional file 8). In contrast to these earlier developmental periods, the adult stage specific differentially expressed genes were relatively less common. The differentially expressed genes of *A. gambiae* at the adult stage (differentially expressed between males and females) included 128 common orthologs among the differentially expressed genes of *A. aegypti,* but only 6 of them were significant between adult males and females of *A. aegypti*. Thus, only ~5% of the orthologous genes reflected adult stage specific expression in both species (Figure 6). These six common genes are related to chromatin assembly and disassembly, protein metabolic process as well as unknown functions. And four of these six genes displayed the same trend in expression in both species (Additional file 8). These results clearly suggest that gene expression patterns during developmental processes may have both common as well as distinct components in the two mosquito species, and also that the expression patterns tend to diverge more in late stages (adult) compared to the earlier stages of development (larvae and pupae).

## Discussion

Although very little is known on molecular aspects of developmental processes of any mosquito, *A. aegypti* is emerging as a model organism for developmental biology studies [37]. The genome sequence of *A. aegypti*[18], along with that for two other mosquito species, *C. quinquefasciatus* (vector of lymphatic filariasis and West Nile virus) [38] and *A. gambiae* (major malaria vector) [39], have greatly enhanced our understanding of several aspects of mosquito biology [40]. A comparative genomic analysis of developmental genes in these mosquitoes with *Drosophila melanogaster*[36] indicated that while orthologs for most *D. melanogaster* developmental genes are present in mosquitoes, some key genes in *D. melanogaster* are not represented. The present investigation was initiated to profile transcriptional changes across the different stages of *A. aegypti* development. Our results represent the first efforts toward uncovering and understanding temporal patterns of gene expression underpinning the processes of larval morphogenesis, pupation and transition to adult stages of *A. aegypti.*

We observed that the majority of DETs (1,173 of 1,551; 75.6%) showed significant differential expression for only a single developmental stage comparison. The differentially expressed genes within life stages were characterized by specific metabolic processes. The earlier stages of the life cycle (larvae and pupae) were significantly associated with KEGG pathway genes related to development, transcription, amino acid metabolism and carbohydrate metabolism, whereas genes related to lipid- and energy-metabolism were significantly associated with the later developmental stages such as pupae-adult transition and between males and females within the adult stage (Additional file 6).

The developmental processes involve several interesting pathways as revealed by analysis of KEGG pathway genes of the differentially expressed transcripts (Figure 5). The genes that were differentially expressed between developmental stages largely represented pathways involved in processing of genetic information such as translation and folding, sorting and degradation of RNA and proteins, and specific metabolisms such as carbohydrate metabolism and energy metabolism as well as several signal transduction processes. The developmental processes of genetic information in *A. aegypti* involved differentially expressed genes related to ribosome, ribosome biogenesis, RNA transport and surveillance of mRNA; these collectively represent different events of protein translation. The post-translational events required for protein folding, sorting and degradation (such as protein processing in the endoplasmic reticulum, proteasome and ubiquitin mediated proteolysis) represented another major component of *A. aegypti* development. Among the carbohydrate and energy metabolism pathways, sugar metabolism, glycolysis, propanoate metabolism and oxidative phosphorylation related genes were among the top-three ranking KEGG pathways represented by the differentially expressed genes. It is long established that energy metabolism is intricately associated with developmental stages of insects [41]. In addition, lipid metabolism (primarily glycerolipid, sphingolipid and fatty acid metabolism) genes were represented by the differentially expressed transcripts between different stages of development. This is consistent with earlier studies that suggest significance of sugar and lipid metabolism in developmental processes of insects [4,22,41-43]. Furthermore, several signal transducing genes were also differentially expressed representing pathways such as Notch signaling, Hedgehog signaling, WNT signaling and others (Additional file 6).

We also observed differential expression of several proteases at different stages of *A. aegypti* development (Additional file 3). Roles for proteases in the developmental process are known in *Xenopus laevis*, specific ciliates and arthropod species [44-46]. During earlier development stages of *A. aegypti*, several genes encoding different types of proteases were significantly differentially expressed but expression changes of the same genes were not significant at the late developmental stages. Two proteasome related genes (AAEL003871 and AAEL007049) were differentially expressed between early pupal to late pupal stages suggesting their possible role in transition from pupal to adult development. Because many proteases have immune related functions in insects and the fact that immunity varies with age [47], it is possible that proteases may have a significant role in the aging processes of the mosquito. Consistent with that, we also identified differentially expressed genes such as AAEL006571 and AAEL010083 which are associated with the Toll and IMD signaling pathways as well as several ras and rab GTPases (AAEL006091, AAEL012071, AAEL013620, AAEL013139) at different stages of *A. aegypti* development.

Genes related to odorant binding (e.g. AAEL003525, AAEL003315 and AAEL006424) were differentially expressed only at the onset of adulthood and are likely associated with development of smell and sense related capabilities necessary for host seeking and other behaviors in newly emerged adults. Adult male and female specific developmental genes were particularly interesting. We found three important gene functions that were significantly associated with differentially expressed genes between males and females. They included genes related to intracellular protein transport (AAEL006091, AAEL003106), vesicle-mediated transport (AAEL003106, AAEL014423) and DNA replication (AAEL012826, AAEL007457, and AAEL010644). Many of these, particularly genes related to actin cytoskeleton organization (AAEL012283), cellular component organization (AAEL012283), receptor activity (AAEL009110) as well as genes related to intracellular protein trafficking (AAEL003106, AAEL006091) play roles in the innate immune response, including response to dengue virus infection [48], in the adult mosquitoes. Because only adult females transmit disease causing pathogens to vertebrates, such as different flaviviruses to vertebrate hosts, differential expression of such genes between males and females may reflect in part their roles in determining vector competence to different pathogens in adult females. Furthermore, application of insecticides such as pyrethroids and organ-ophosphates is routinely practiced to control of *A. aegypti* larval and adult, respectively. Research suggests that resistance developed to these compounds can have confounding effects on development of *A. aegypti*[12].

A comparative genomic analysis between *D. melanogaster* and mosquito developmental pathways identified several key genes that reflect conserved developmental processes in mosquitoes as well fruit flies [36]. Two particular genes, 14-3-3zeta (AAEL006885) and modifier of mdg4 (AAEL010576), which are conserved as 1:1 orthologs between *D. melanogaster* and *A. aegypti* (also represented as single copy orthologs in the *A. gambiae* and *C. quinquefasciatus* genomes) were significantly up-regulated during the transition from early larval to late larval stages of the mosquito. Identification of these genes and pathways related to development implies key roles for these genes in evolution of development within mosquitoes and fruit flies. At the same time, several key genes that are known to play roles in the development of fruit flies were not identified from our study. That can be attributed not only to the fact that we utilized custom cDNA microarrays that do not represent all the annotated genes in *A. aegypti*, but may also be due in part to extensive divergence of many developmental genes within dipterans [36,49-51].

In regard to comparison with the *A. gambiae* developmental transcriptome, our results suggest that conservation of gene expression between the two species decreases as the mosquitoes develop to later developmental stages. The percentage of genes that are conservatively expressed during adult stage is ~5-fold less compared to that we observed at the larval stages between the two mosquitoes. It is likely that many of the genetic components related to developmental processes have undergone evolutionary changes between the two species.

## Conclusions

This is the first report on an effort to characterize the developmental transcriptome of *A. aegypti*. Our results show that genes involved in the developmental programs of this mosquito are highly stage-specific and that the molecular events associated with transitions through the larval, pupal and adult stages are largely discrete. Comparison with the *A. gambiae* developmental transcriptome suggests that gene expression during developmental processes reflects both common as well as distinct patterns between the two mosquito species.

## Competing interest

The authors declared that they have no competing interest.

## Authors’ contributions

BWH and SKB performed the experiments, contributed to data analysis, and helped in drafting the manuscript; BSD, DDL and AM contributed reagents and materials for the microarrays; JRS helped in designing the study and data analysis; DWS conceived the project, helped in designing the study, and helped in drafting the manuscript. All authors read and approved of the final version.

## Supplementary Material

Additional file 1 List of primers used for qRT-PCR. Click here for file

Additional file 2 **Comparison of qRT-PCR and microarray expression data for a subset of randomly selected genes.** The cDNA clone ID and the VectorBase gene ID corresponding to these DETs are as follows: NAAFC38 (AAEL000101), NABOS06 (AAEL004371), NABPX34 (AAEL003461), NACAR71 (AAEL011290), NACAW66 (AAEL008664), NADBA22 (AAEL010048), NADC788 (AAEL007839), NADED04 (AAEL001397), NADWY24 (AAEL000678). Click here for file

Additional file 3 **List of annotated genes differentially expressed at specific developmental stages in *Aedes aegypti.*** The gene ID and gene description are shown for each group. These groups correspond to the expression clusters shown in Figure 2. Click here for file

Additional file 4 **List of annotated genes differentially expressed in overlapping developmental stages of *Aedes aegypti.*** Click here for file

Additional file 5 **Significant association of GO terms with DETs at different stages of *Aedes aegypti *development.** The numbers of genes specific/non-specific to each stage associated/not-associated with specific GO terms are shown. Fisher’s exact test p-values for significant association are also shown. Click here for file

Additional file 6 **Significant associations between stage-specific DETs and KEGG pathways.** The numbers shown are the counts of genes of each category shown in first row that corresponds to the significant pathways (shown in first column) at specific developmental stages (shown in second column). The Fisher’s exact test p-values of significance are shown in each case. Click here for file

Additional file 7 Differential expression of signaling genes. Click here for file

Additional file 8 **Comparison of gene expression patterns for *Aedes aegypti *with the orthologous genes in *Anopheles gambiae *at larvae (8-A), larvae to pupae transition (8-B) and adult stage (male versus female) (8-C).** The *A. gambiae* microarray results were obtained from a previously reported study [22]. Click here for file
