# Supplementary material for: Stage-specific transcription during development of Aedes aegypti
Source: BMC Dev Biol. 2013 Jul 22;13:29. doi: 10.1186/1471-213X-13-29 (PMC3728235; doi:10.1186/1471-213X-13-29)
Supplement: Additional file 1 — List of primers used for qRT-PCR. [file 1471-213X-13-29-S1.docx]

List of primers used in the qRT-PCR assays.

| Gene | Forward | Reverse |
| --- | --- | --- |
| AAEL000101 | CCCATTGTCCTTCACGGATTT | CAATCCTTGCCCAGGTTGAT |
| AAEL004371 | GTAGGAGATTGCCGTGACAAATT | CGGAAGACTTCGCCATTAAACT |
| AAEL003461 | CACCGTAGCCGACCAGATG | CGGACTTCCTTACGTGGAAAAC |
| AAEL011290 | CCACTGCCGCCTTTCAAC | TTTTACGCGCGGTACGATTT |
| AAEL008664 | GGGTTCTCAATCTGGACGAAAT | TCGACCAAATCCGAGGATATCT |
| AAEL010048 | CCGGACGGTTGGTTTCC | GCTGGTCGTCTCCATTGCA |
| AAEL007839 | ACGTCAAGGGCTGGAAGAACT | CGCCGGATCGGTCAAA |
| AAEL001397 | CAGGATCGACTTCCGTTGGA | GGATGACCAATGGCTTGCTT |
| AAEL000678 | CCACGGTTTGGAATAGTTTGTG | CCTGCTCGCACCCCATT |
