# Supplementary figures and images for: Stage-specific transcription during development of Aedes aegypti
Source: BMC Dev Biol. 2013 Jul 22;13:29. doi: 10.1186/1471-213X-13-29 (PMC3728235; doi:10.1186/1471-213X-13-29)

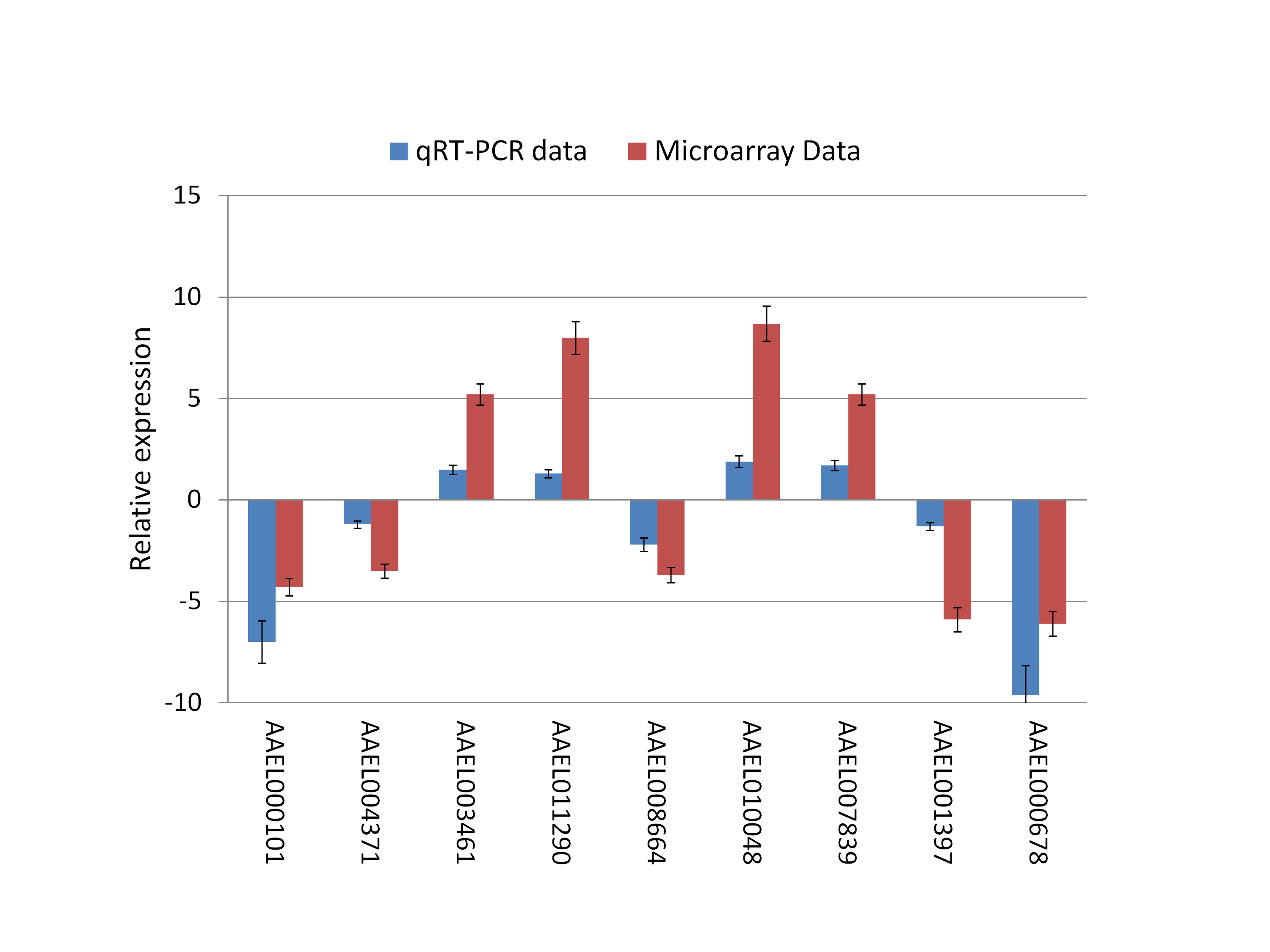

Supplement: Additional file 2 — Comparison of qRT-PCR and microarray expression data for a subset of randomly selected genes. The cDNA clone ID and the VectorBase gene ID corresponding to these DETs are as follows: NAAFC38 (AAEL000101), NABOS06 (AAEL004371), NABPX34 (AAEL003461), NACAR71 (AAEL011290), NACAW66 (AAEL008664), NADBA22 (AAEL010048), NADC788 (AAEL007839), NADED04 (AAEL001397), NADWY24 (AAEL000678). [file 1471-213X-13-29-S2.tiff]
