# Supplementary material for: Stage-specific transcription during development of Aedes aegypti
Source: BMC Dev Biol. 2013 Jul 22;13:29. doi: 10.1186/1471-213X-13-29 (PMC3728235; doi:10.1186/1471-213X-13-29)
Supplement: Additional file 5 — Significant association of GO terms with DETs at different stages of Aedes aegypti development. The numbers of genes specific/non-specific to each stage associated/not-associated with specific GO terms are shown. Fisher’s exact test p-values for significant association are also shown. [file 1471-213X-13-29-S5.docx]

Significant association of specific GO terms with transcripts differentially expressed at different stages of *A. aegypti* development. The numbers of genes specific/ non-specific to each stage associated/not-associated with specific GO terms are shown (see foot notes for detailed explanation).

| GO # | GO term name | Developmental stages | GO_total | GO_stage specific | GO_stage non-specific | Other GO_stage specific | Other GO_stage non-specific | p-value |
| --- | --- | --- | --- | --- | --- | --- | --- | --- |
| 1 | isomerase activity | EL-LL | 66 | 3 | 1 | 337 | 1385 | 0.026 |
| 2 | serine-type endopeptidase inhibitor activity | EL-LL | 103 | 3 | 1 | 337 | 1385 | 0.026 |
| 3 | iron-sulfur cluster binding | LL-EP | 110 | 2 | 1 | 113 | 1609 | 0.013 |
| 4 | tricarboxylic acid cycle | LL-EP | 120 | 2 | 1 | 113 | 1608 | 0.013 |
| 5 | hydrolase activity | LL-EP | 7 | 7 | 39 | 108 | 1570 | 0.030 |
| 6 | metalloendopeptidase activity | LL-EP | 77 | 2 | 3 | 113 | 1608 | 0.039 |
| 7 | structural molecule activity | LL-EP | 57 | 2 | 3 | 113 | 1606 | 0.039 |
| 8 | protein binding | EP-LP | 1 | 54 | 52 | 1025 | 595 | 0.013 |
| 9 | transferase activity | EP-LP | 21 | 13 | 18 | 1066 | 629 | 0.023 |
| 10 | iron ion binding | EP-LP | 44 | 8 | 0 | 1071 | 647 | 0.029 |
| 11 | receptor activity | EP-LP | 45 | 3 | 7 | 1076 | 640 | 0.047 |
| 12 | protein binding | LP-AdultMix | 1 | 15 | 91 | 117 | 1503 | 0.021 |
| 13 | transferase activity | LP-AdultMix | 21 | 6 | 25 | 126 | 1569 | 0.027 |
| 14 | serine-type endopeptidase activity | LP-AdultMix | 25 | 5 | 16 | 127 | 1578 | 0.018 |
| 15 | odorant binding | LP-AdultMix | 54 | 2 | 2 | 130 | 1592 | 0.031 |
| 16 | intracellular protein transport | AM-AF | 37 | 2 | 7 | 58 | 1659 | 0.037 |
| 17 | vesicle-mediated transport | AM-AF | 59 | 2 | 4 | 58 | 1662 | 0.016 |
| 18 | DNA replication | AM-AF | 65 | 3 | 3 | 57 | 1663 | 0.001 |

* The numbers under the column “GO_total” represent the number of transcripts associated with the indicated GO term irrespective of their differential expression between the developmental stages investigated. The numbers under the column “GO_stage specific” represent the number of transcripts associated with the indicated GO term and at the same time differentially expressed between the indicated developmental stages. The numbers under the column “GO_stage non-specific” represent the number of transcripts associated with the indicated GO term and they are differentially expressed between the developmental stages other than the indicated ones. The numbers under the column “Other GO_stage specific” represent the number of transcripts associated with GO terms other than the indicated term, but they are differentially expressed between the indicated developmental stages. These four counts of transcripts, in each row, were used to calculate the Fisher’s exact test p-values for significant association of DETs with the specific GO terms. The numbers under the column “Other GO_stage specific” are not used to calculate the *p*-value but are provided to show the total number of DETs identified from the array data that were neither associated with the indicated GO term nor differentially expressed between the indicated developmental stages.
