# Supplementary material for: Stage-specific transcription during development of Aedes aegypti
Source: BMC Dev Biol. 2013 Jul 22;13:29. doi: 10.1186/1471-213X-13-29 (PMC3728235; doi:10.1186/1471-213X-13-29)
Supplement: Additional file 6 — Significant associations between stage-specific DETs and KEGG pathways. The numbers shown are the counts of genes of each category shown in first row that corresponds to the significant pathways (shown in first column) at specific developmental stages (shown in second column). The Fisher’s exact test p-values of significance are shown in each case. [file 1471-213X-13-29-S6.docx]

Significant association between stage-specific DETs and KEGG pathways. The numbers shown are the counts of genes of each category shown in first row corresponding to specific pathways (shown in first column) at specific developmental stages (shown in second column). The Fisher exact test p-values of significances are shown in each case.

| Specific pathway | Specific stage | Pathway and stage specific genes | Pathway specific but stage non-specific genes | Stage specific but pathway non-specific genes | Genes not specific to the pathway or stage | p-value |
| --- | --- | --- | --- | --- | --- | --- |
| Development | EL-LL | 2 | 0 | 69 | 286 | 0.039 |
| Metabolism of Other Amino Acids | EL-LL | 3 | 2 | 68 | 283 | 0.050 |
| Translation | LL-EP | 4 | 45 | 67 | 239 | 0.033 |
| Translation | EP-LP | 24 | 25 | 47 | 239 | 0.000 |
| Transcription | EP-LP | 8 | 6 | 63 | 274 | 0.002 |
| Folding, Sorting and Degradation | EP-LP | 19 | 31 | 52 | 238 | 0.002 |
| Carbohydrate Metabolism | EP-LP | 14 | 20 | 57 | 254 | 0.003 |
| Transport and Catabolism | EP-LP | 8 | 10 | 63 | 270 | 0.015 |
| Amino Acid Metabolism | EP-LP | 8 | 10 | 63 | 270 | 0.015 |
| Glycan Biosynthesis and Metabolism | EP-LP | 6 | 7 | 65 | 275 | 0.028 |
| Energy Metabolism | LP-AdultMix | 8 | 13 | 63 | 267 | 0.048 |
| Lipid Metabolism | LP-AdultMix | 5 | 6 | 66 | 277 | 0.048 |
| Translation | AM-FM | 2 | 47 | 69 | 239 | 0.002 |
| Folding, Sorting and Degradation | AM-FM | 3 | 47 | 68 | 238 | 0.007 |
