# Supplementary material for: Stage-specific transcription during development of Aedes aegypti
Source: BMC Dev Biol. 2013 Jul 22;13:29. doi: 10.1186/1471-213X-13-29 (PMC3728235; doi:10.1186/1471-213X-13-29)
Supplement: Additional file 7 — Differential expression of signaling genes. [file 1471-213X-13-29-S7.docx]

**Differential expression of signaling genes during *A. aegypti* development.**

| Gene | Pathway | Differential expression |
| --- | --- | --- |
| AAEL009110 | TGF-beta signaling pathway | AM-AF |
| AAEL011325 | Neuroactive ligand-receptor interaction | EL-LL |
| AAEL004586 | Notch signaling pathway | EL-LL |
| AAEL005515 | Dorso-ventral axis formation | EL-LL |
| AAEL007372 | Neuroactive ligand-receptor interaction | EL-LL |
| AAEL004586 | Notch signaling pathway | EP-LP |
| AAEL004351 | Hedgehog signaling pathway | EP-LP |
