# Supplementary material for: Stage-specific transcription during development of Aedes aegypti
Source: BMC Dev Biol. 2013 Jul 22;13:29. doi: 10.1186/1471-213X-13-29 (PMC3728235; doi:10.1186/1471-213X-13-29)
Supplement: Additional file 8 — Comparison of gene expression patterns for Aedes aegypti with the orthologous genes in Anopheles gambiae at larvae (8-A), larvae to pupae transition (8-B) and adult stage (male versus female) (8-C). The A. gambiae microarray results were obtained from a previously reported study [22]. [file 1471-213X-13-29-S8.docx]

Additional file 8-A

1: AAEL010576/AGAP003439, 2: AAEL010048/AGAP002061, 3: AAEL010048/AGAP002061, 4: AAEL007765/AGAP005246, 5: AAEL007765/AGAP005246, 6: AAEL006885/AGAP007643, 7: AAEL001865/AGAP007780, 8: AAEL001865/AGAP007780, 9: AAEL008192/AGAP001910, 10: AAEL002978/AGAP003475, 11: AAEL008192/AGAP001910, 12: AAEL002881/AGAP003325, 13: AAEL002216/AGAP008195, 14: AAEL004873/AGAP004551, 15: AAEL008192/AGAP001910, 16: AAEL010048/AGAP002061, 17: AAEL010048/AGAP002061, 18: AAEL010777/AGAP009584, 19: AAEL010777/AGAP005462, 20: AAEL010576/AGAP003685, 21: AAEL007765/AGAP005246, 22: AAEL000271/AGAP006670, 23: AAEL007765/AGAP005246, 24: AAEL010777/AGAP003338, 25: AAEL004873/AGAP004551, 26: AAEL007765/AGAP005246, 27: AAEL014863/AGAP007724, 28: AAEL004873/AGAP004551, 29: AAEL004565/AGAP002931

Additional file 8-B

1: AAEL010048/AGAP002061, 2: AAEL010048/AGAP004953, 3: AAEL003427/AGAP011424, 4: AAEL003427/AGAP011424, 5: AAEL003746/AGAP004396, 6: AAEL007822/AGAP002169, 7: AAEL006158/AGAP001813, 8: AAEL010048/AGAP002061, 9: AAEL010048/AGAP004953, 10: AAEL003427/AGAP011424, 11: AAEL009029/AGAP003652, 12: AAEL006929/AGAP003605, 13: AAEL004235/AGAP000159, 14: AAEL003746/AGAP004163, 15: AAEL004565/AGAP002931

Additional file 8-C

1: AAEL010576/AGAP003439, 2: AAEL011157/AGAP003900, 3: AAEL011098/AGAP003227, 4: AAEL007501/AGAP002933, 5: AAEL010576/AGAP003685, 6: AAEL004565/AGAP002931
